# Prediction of Post-hepatectomy Liver Failure in Patients With Hepatocellular Carcinoma Based on Radiomics Using Gd-EOB-DTPA-Enhanced MRI: The Liver Failure Model

**DOI:** 10.3389/fonc.2021.605296

**Published:** 2021-03-10

**Authors:** Yuyan Chen, Zelong Liu, Yunxian Mo, Bin Li, Qian Zhou, Sui Peng, Shaoqiang Li, Ming Kuang

**Affiliations:** ^1^Department of Liver Surgery, The First Affiliated Hospital of Sun Yat-sen University, Guangzhou, China; ^2^Division of Interventional Ultrasound, The First Affiliated Hospital of Sun Yat-sen University, Guangzhou, China; ^3^State Key Laboratory of Oncology in South China, Department of Radiology, Collaborative Innovation Center for Cancer Medicine, Sun Yat-sen University Cancer Center, Guangzhou, China; ^4^Clinical Trial Unit, The First Affiliated Hospital of Sun Yat-sen University, Guangzhou, China; ^5^Department of Gastroenterology and Hepatology, The First Affiliated Hospital of Sun Yat-sen University, Guangzhou, China

**Keywords:** prediction model, magnetic resonance imaging, radiomics, post-hepatectomy liver failure, hepatocellular carcinoma

## Abstract

**Objectives:** Preoperative prediction of post-hepatectomy liver failure (PHLF) in patients with hepatocellular carcinoma (HCC) is significant for developing appropriate treatment strategies. We aimed to establish a radiomics-based clinical model for preoperative prediction of PHLF in HCC patients using gadolinium-ethoxybenzyl-diethylenetriamine (Gd-EOB-DTPA)-enhanced magnetic resonance imaging (MRI).

**Methods:** A total of 144 HCC patients from two medical centers were included, with 111 patients as the training cohort and 33 patients as the test cohort, respectively. Radiomics features and clinical variables were selected to construct a radiomics model and a clinical model, respectively. A combined logistic regression model, the liver failure (LF) model that incorporated the developed radiomics signature and clinical risk factors was then constructed. The performance of these models was evaluated and compared by plotting the receiver operating characteristic (ROC) curve and calculating the area under the curve (AUC) with 95% confidence interval (CI).

**Results:** The radiomics model showed a higher AUC than the clinical model in the training cohort and the test cohort for predicting PHLF in HCC patients. Moreover, the LF model had the highest AUCs in both cohorts [0.956 (95% CI: 0.955–0.962) and 0.844 (95% CI: 0.833–0.886), respectively], compared with the radiomics model and the clinical model.

**Conclusions:** We evaluated quantitative radiomics features from MRI images and presented an externally validated radiomics-based clinical model, the LF model for the prediction of PHLF in HCC patients, which could assist clinicians in making treatment strategies before surgery.

## Introduction

Hepatocellular carcinoma (HCC) is one of the most common malignancies and the fourth leading cause of cancer death worldwide (1). Surgical resection is an effective curative treatment for HCC patients, which provides remarkable survival benefits (2). However, the postoperative mortality of hepatectomy is estimated to be about 3–14% (3), which is even higher in patients with preexisting chronic liver disease (4). The main reason for the high mortality is postoperative complications, including bleeding, incisional infection, and liver failure, etc. Among them, post-hepatectomy liver failure (PHLF) is a severe one and the major cause of death (4, 5), with an incidence of 12% (6, 7) and a mortality of up to 50% (3). PHLF could also result in prolonged hospitalization, increased costs, and poor long-term prognosis (3). Therefore, the risk of PHLF needs to be assessed accurately before hepatectomy.

Previous literature has reported some clinical factors that contributed to PHLF in HCC patients, including tumor size (8), preoperative platelet (PLT) count (4, 9), future liver remnant (FLR) (10), etc. Currently, commonly used clinical methods for preoperative liver function assessment include routine blood test, blood biochemistry test, indocyanine green (ICG) retention test, and clinical scores such as Child-Pugh (CP) score, model for end-stage liver disease (MELD) score, and albumin-bilirubin (ALBI) score (11–13). However, all these methods are limited to some extent with unsatisfactory performance, which is probably due to limitations of clinical variables. Therefore, there is an urgent need to explore a more effective means of predicting PHLF.

In recent years, as radiomics has evolved rapidly, it becomes increasingly promising in medical research. Quantitative features could be extracted from digital medical images using radiomics so that these high-dimensional data can be fully used for assisting clinicians in disease diagnosis, treatment strategy development, and prognosis assessment. Several studies have shown that texture features were significantly associated with overall survival (OS) and recurrence-free survival (RFS) of HCC patients after hepatectomy (14, 15). It has also been found that radiomics features were associated with liver fibrosis and other pathologic features of HCC (16, 17). Therefore, it is promising to apply radiomics to further assess the liver function of HCC patients after hepatectomy. However, there is a lack of study that researches the relationship between radiomics and PHLF.

In this study, we aimed to establish models for the prediction of PHLF in HCC patients using radiomics based on gadolinium-ethoxybenzyl-diethylenetriamine (Gd-EOB-DTPA)-enhanced magnetic resonance imaging (MRI), which could potentially assist doctors in clinical decision making.

## Materials and Methods

### Patients

The retrospective study was conducted in two medical centers: First Affiliated Hospital of Sun Yat-sen University (FAHSYSU) and Sun Yat-sen University Cancer Center (SYSUCC). Patients from FASHSYSU were used as the training cohort for model development, while patients from SYSUCC were used as the test cohort for model validation. Patients who were diagnosed with HCC from January 2016 to December 2019 were screened. Inclusion criteria were: (1) underwent hemihepatectomy; (2) pathologically diagnosed with HCC; (3) received Gd-EOB-DTPA-enhanced MRI scan of the liver and ICG retention test within 30 days prior to surgery. Exclusion criteria were: (1) received any anti-tumor therapy before the surgery; (2) incomplete clinical or pathological information. In accordance with the International Study Group of Liver Surgery (ISGLS), PHLF is defined as impaired functions of the liver, which are characterized by hyperbilirubinemia and an increased international normalized ratio (INR) on or after postoperative day 5 (18).

### Clinical Variables

Clinical variables including gender, age, height, weight, body mass index (BMI), diabetes mellitus (DM), history of alcohol consumption, non-alcoholic fatty liver disease (NAFLD), viral hepatitis (VH), hepatic encephalopathy (HE), ascites, performance status (PS) score, CP score, Barcelona clinic liver cancer (BCLC) stage, MELD score, and ALBI score were collected from medical records. Laboratory test results within 7 days prior to surgery including PLT count, total bilirubin (TBIL), serum creatinine (Cr), INR, alanine aminotransferase (ALT), aspartate aminotransferase (AST), γ-glutamyl transpeptidase (GGT), and ICG retention test at 15 min (ICG-R15) were obtained. BMI was determined by dividing the weight (kg) by the square of the height (cm). The CP score was determined by five parameters: serum albumin, TBIL, prothrombin time, hepatic encephalopathy, and ascites (19). The MELD score was calculated by using the formula: 3.8 × ln [TBIL (mg/dL)] + 11.2 × ln (INR) + 9.6 × ln [Cr (mg/dL)] + 6.4 (20). The equation for the ALBI score calculation was 0.66 × log_10_ [TBIL (μmol/L)] – 0.085 × [albumin (g/L)] (21).

### MRI Image Acquisition and Evaluation

Details of MRI image acquisition are provided in Supplementary Materials and Supplementary Table 1.

Three independent radiologists with more than 20 years of experience reviewed the MRI images and evaluate the following features: transient hepatic parenchymal enhancement (THPE), tumor size, number of tumors, tumor boundary, tumor capsule, vascular invasion, bile duct invasion, bile duct dilatation, lymph node metastasis, adjacent tissue invasion, varicose veins, hemorrhage, tumor thrombus, liver cirrhosis, and splenomegaly.

### Region-of-Interest Segmentation and Radiomics Feature Extraction

**Figure 1:**
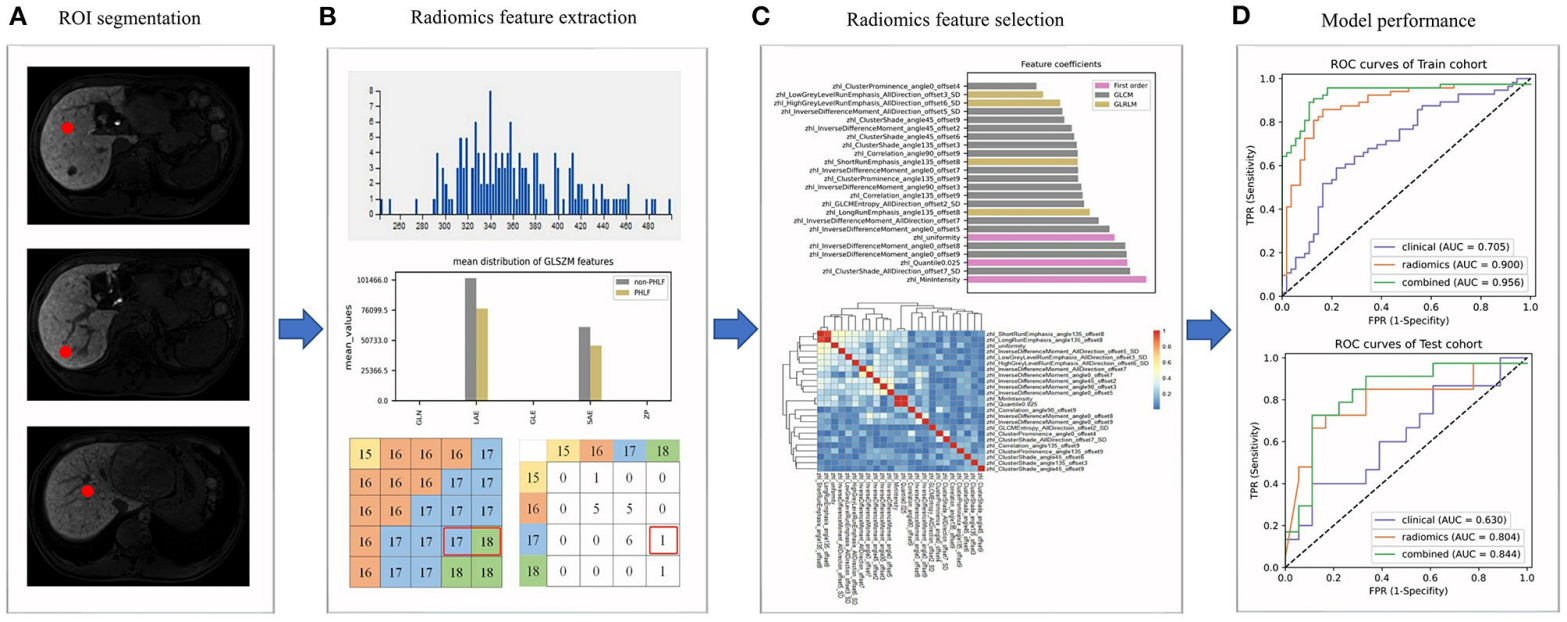
The radiomics workflow. **(A)** Regions of interests (ROIs) were delineated in the non-tumor area; **(B)** Radiomics features were extracted from ROIs; **(C)** The selection of radiomics features; **(D)** The evaluation of the performance of prediction models.

The radiomics workflow is shown in Figure 1. MRI images of patients were imported into the ITK-SNAP 3.6.0 software (open-source software; www.itksnap.org) (22). By using the software, the radiologists delineated a circular region of interest (ROI) of 1 cm in diameter in each non-tumor liver segment as indicated in the hepatobiliary phase on the transverse slice.

We then extracted and analyzed the MRI image features by using the A.K. 2.0.0 software (house-made software; Analysis-Kit, GE Healthcare). In total, 1,044 MRI image features of five categories were extracted, including seven shape features, 44 first order histogram features, 61 gray level size zone matrix (GLSZM) features, 446 gray level cooccurrence matrix (GLCM) features, and 486 gray level run-length matrix (GLRLM) features.

### Development and Validation of the Radiomics Model

The feature analysis was performed by using open-source software (https://github.com/salan668/FAE). Original values of radiomics features were normalized, where each value subtracted the mean and then was divided by the L2 norm. Pearson's correlation coefficients of 1,044 radiomics features in each patient were then calculated. Radiomics features with a correlation coefficient higher than 0.86 were considered highly correlated and would be randomly eliminated, leaving only one. The recursive feature elimination (RFE) method was used for subsequent feature selection. It is much more robust to data over-fitting than other feature selection techniques and has shown its power in many fields including radiomics, genomics, proteomics, and metabolomics (23). The best features were picked out from the whole by repeatedly constructing the model, and then remaining features were used to select the best features. This process was repeated until all features were traversed and the order in which features were eliminated in the process was the ordering of features. Thus, each feature was evaluated on their contribution to the model. After selection, a desired number of features were included in a logistic regression model. Finally, five-fold cross-validation was used to prevent overfitting.

Based on the radiomics model, a radiomics score that indicated the relative risk of PHLF for each patient in both the training cohort and the test cohort was calculated. The actual PHLF was determined by clinical evaluation as mentioned before. Then the performance of the radiomics model in each cohort was evaluated by plotting the receiver operating characteristic (ROC) curve and calculating the area under the curve (AUC), accuracy, sensitivity, specificity, positive predictive value (PPV), and negative predictive value (NPV).

### Development and Validation of the Clinical Model and the Combined Model

In the training cohort, clinical variables were included in the univariate logistic regression analysis. The odds ratios (ORs) and corresponding 95% confidence intervals (CIs) for all variables were calculated. Each variable with a *p*-value lower than 0.2 was further included in the multivariate logistic regression analysis, in which the corrected ORs and the corresponding 95% CIs were calculated. A variable with a *p*-value lower than 0.05 was considered as an independent risk factor for PHLF.

Based on the selected clinical features, a clinical model was then established. After combining with the radiomics signature, a combined model called the liver failure (LF) model was then constructed. Five-fold cross-validation was used for the model's tuning. The performance of both models in each cohort was evaluated like the radiomics model.

### Statistical Analysis

Categorical variables were compared by using Chi-square tests or Fisher's exact tests. For continuous variables, Kolmogorov–Smirnov tests and Levene tests were firstly used for testing normality of distribution and homogeneity of variance, respectively. Student's *t*-test was used if the variable satisfied both normal distribution and homogeneous variance, otherwise Mann–Whitney *U*-test was used. All statistical tests were two-tailed. Python 3.6 software was used for all statistical analyses. Graphs were generated by using the matplotlib package and the seaborn package in python 3.6 software and the pheatmap package in R 3.4 software.

## Results

### Patient Characteristics

A total of 144 HCC patients were included in the study, among whom 111 were from FAHSYSU (training cohort) and 33 were from SYSUCC (test cohort). For the training cohort, the number of PHLF and non-PHLF patients were 56 and 55, respectively. For the test cohort, the number of PHLF and non-PHLF patients were 15 and 18, respectively. Clinical characteristics of patients in two cohorts were shown and compared in Table 1.

**Table 1:** Baseline characteristics of the FAHSYSU cohort and the SYSUCC cohort.

**Variables**	**Levels**	**FAHSYSU cohort**				**SYSUCC cohort**				***P-*value**
		**Total (*n* = 111)**	**PHLF (*n* = 56)**	**Non PHLF (*n* = 55)**	***P-*value**	**Total (*n* = 33)**	**PHLF (*n* = 15)**	**Non PHLF (*n* = 18)**	***P-*value**	
Age	Median (IQR)	54.00 (17.00)	54.50 (18.00)	53.00 (17.00)	0.552	50.00 (12.00)	55.00 (12.00)	48.00 (13.25)	0.354	0.637
Gender	Male (%)	97 (87.39%)	51 (91.07%)	46 (83.64%)	0.267	29 (87.88%)	11 (73.33%)	18 (100.00%)	0.033	1.000
	Female (%)	14 (12.61%)	5 (8.93%)	9 (16.36%)		4 (12.12%)	4 (26.67%)	0 (0.00%)		
Height	Median (IQR)	167.00 (7.00)	168.00 (7.00)	167.00 (9.50)	0.132	170.00 (12.00)	168.00 (11.50)	172.00 (13.50)	0.069	0.216
Weight	Median (IQR)	62.00 (14.75)	62.00 (15.25)	61.00 (13.25)	0.423	64.00 (10.00)	66.50 (10.00)	63.75 (13.25)	0.908	0.147
BMI	Median (IQR)	22.23 (3.75)	22.45 (3.98)	22.05 (3.39)	1.000	22.76 (3.99)	23.23 (4.43)	22.33 (2.52)	0.317	0.217
DM	Absent (%)	99 (89.19%)	50 (89.29%)	49 (89.09%)	1.000	29 (87.88%)	14 (93.33%)	15 (83.33%)	0.607	0.762
	Present (%)	12 (10.81%)	6 (10.71%)	6 (10.91%)		4 (12.12%)	1 (6.67%)	3 (16.67%)		
Alcohol consumption	Absent (%)	79 (71.17%)	41 (73.21%)	38 (69.09%)	0.679	22 (66.67%)	11 (73.33%)	11 (61.11%)	0.712	0.667
	Present (%)	32 (28.83%)	15 (26.79%)	17 (30.91%)		11 (33.33%)	4 (26.67%)	7 (30.91%)		
NAFLD	Absent (%)	109 (98.20%)	55 (98.21%)	54 (98.18%)	1.000	33 (100.00%)	15 (100.00%)	18 (100.00%)	1.000	1.000
	Present (%)	2 (1.80%)	1 (1.79%)	1 (1.82%)		0 (0.00%)	0 (0.00%)	0 (0.00%)		
VH	HBV (%)	89 (80.18%)	49 (87.50%)	40 (72.73%)	0.060	22 (66.67%)	9 (60.00%)	13 (72.22%)	0.488	0.155
	HCV (%)	0 (0.00%)	0 (0.00%)	0 (0.00%)		0 (0.00%)	0 (0.00%)	0 (0.00%)		
	Neither (%)	22 (19.82%)	7 (12.50%)	15 (27.27%)		11 (33.33%)	6 (40.00%)	5 (27.78%)		
	Both (%)	0 (0.00%)	0 (0.00%)	0 (0.00%)		0 (0.00%)	0 (0.00%)	0 (0.00%)		
CP score	A (%)	111 (100.00%)	56 (100.00%)	55 (100.00%)	1.000	33 (100.00%)	15 (100.00%)	18 (100.00%)	1.000	1.000
	B (%)	0 (0.00%)	0 (0.00%)	0 (0.00%)		0 (0.00%)	0 (0.00%)	0 (0.00%)		
	C (%)	0 (0.00%)	0 (0.00%)	0 (0.00%)		0 (0.00%)	0 (0.00%)	0 (0.00%)		
BCLC stage	0~A (%)	53 (47.75%)	25 (44.64%)	28 (50.91%)	0.795	31 (93.94%)	14 (93.33%)	17 (94.44%)	1.000	<0.000
	B (%)	20 (18.02%)	11 (19.64%)	9 (16.36%)		2 (6.06%)	1 (6.67%)	1 (5.56%)		
	C (%)	38 (34.23%)	20 (35.71%)	18 (32.73%)		0 (0.00%)	0 (0.00%)	0 (0.00%)		
MELD score	Median (IQR)	6.87 (1.07)	7.11 (1.49)	6.71 (5.22)	0.010	6.43 (6.49)	6.43 (6.97)	6.43 (4.21)	0.818	0.070
ALBI score	Median (IQR)	0.69 (0.19)	0.70 (0.20)	0.66 (0.19)	0.477	0.61 (0.19)	0.64 (0.20)	0.59 (0.21)	0.044	0.025
PS score	Normal (%)	111 (100.00%)	56 (100.00%)	55 (100.00%)	1.000	33 (100.00%)	15 (100.00%)	18 (100.00%)	1.000	1.000
	Abnormal (%)	0 (0.00%)	0 (0.00%)	0 (0.00%)		0 (0.00%)	0 (0.00%)	0 (0.00%)		
ICG-R15	Median (IQR)	6.70 (4.57)	7.45 (4.10)	6.15 (5.20)	0.293	2.90 (2.80)	1.80 (0.97)	3.05 (0.80)	0.591	0.000
Ascites	Absent (%)	111 (100.00%)	56 (100.00%)	55 (100.00%)	1.000	33 (100.00%)	15 (100.00%)	18 (100.00%)	1.000	1.000
	Present (%)	0 (0.00%)	0 (0.00%)	0 (0.00%)		0 (0.00%)	0 (0.00%)	0 (0.00%)		
HE	Absent (%)	111 (100.00%)	56 (100.00%)	55 (100.00%)	1.000	33 (100.00%)	15 (100.00%)	18 (100.00%)	1.000	1.000
	Present (%)	0 (0.00%)	0 (0.00%)	0 (0.00%)		0 (0.00%)	0 (0.00%)	0 (0.00%)		
TBIL	Median (IQR)	14.30 (7.70)	14.55 (7.82)	13.60 (7.25)	0.494	12.30 (5.90)	12.70 (7.82)	11.75 (7.65)	0.062	0.082
Cr	Median (IQR)	76.00 (21.00)	76.50 (23.25)	75.00 (19.50)	0.363	77.90 (16.80)	77.70 (29.15)	78.65 (15.45)	0.829	0.727
INR	Median (IQR)	1.02 (0.10)	1.04 (0.10)	0.99 (0.10)	0.003	1.01 (0.11)	1.00 (0.12)	1.01 (0.10)	0.670	0.725
AST	Median (IQR)	45.00 (33.00)	49.00 (30.50)	41.00 (33.50)	0.067	29.60 (14.50)	33.20 (23.45)	25.30 (9.73)	0.065	0.000
ALT	Median (IQR)	36.00 (30.00)	38.50 (31.50)	35.00 (24.50)	0.286	36.20 (32.20)	37.10 (20.75)	25.15 (32.32)	0.240	0.560
GGT	Median (IQR)	106.50 (157.75)	125.50 (141.75)	102.00 (139.25)	0.175	84.80 (109.90)	92.30 (104.15)	72.75 (108.40)	0.527	0.089
PLT	Median (IQR)	205.00 (112.50)	199.00 (132.00)	208.00 (117.00)	0.215	216.00 (89.00)	224.00 (48.00)	202.50 (107.25)	0.731	0.384
Tumor size	Median (IQR)	7.60 (5.05)	9.55 (4.70)	6.90 (4.65)	0.002	5.80 (4.60)	7.00 (5.10)	5.10 (4.23)	0.270	0.014
Number of tumors	1 (%)	74 (66.67%)	37 (66.07%)	37 (67.27%)	1.000	30 (90.91%)	14 (93.33%)	16 (88.89%)	1.000	0.007
	2 (%)	11 (9.91%)	6 (10.71%)	5 (9.09%)		3 (9.09%)	1 (6.67%)	2 (11.11%)		
	>3 (%)	26 (23.42%)	13 (23.22%)	13 (23.64%)		0 (0.00%)	0 (0.00%)	0 (0.00%)		
Vascular invasion	Absent (%)	73 (65.77%)	36 (64.29%)	37 (67.27%)	0.842	29 (87.88%)	14 (93.33%)	15 (83.33%)	0.607	0.016
	Present (%)	38 (34.23%)	20 (35.71%)	18 (32.73%)		4 (12.12%)	1 (6.67%)	3 (16.67%)		
Splenomegaly	Absent (%)	93 (83.78%)	44 (78.57%)	49 (89.09%)	0.198	27 (81.82%)	13 (86.67%)	14 (77.78%)	0.665	0.793
	Present (%)	18 (16.22%)	12 (21.43%)	6 (10.91%)		6 (18.18%)	2 (13.33%)	4 (22.22%)		
Varicose veins	Absent (%)	100 (90.09%)	50 (89.29%)	50 (90.91%)	1.000	32 (96.97%)	14 (93.33%)	18 (100.00%)	0.455	0.297
	Present (%)	11 (9.91%)	6 (10.71%)	5 (9.09%)		1 (3.03%)	1 (6.67%)	0 (0.00%)		
Adjacent tissue invasion	Absent (%)	109 (98.20%)	56 (100.00%)	53 (96.36%)	0.243	31 (93.94%)	15 (100.00%)	16 (88.89%)	0.489	0.225
	Present (%)	2 (1.80%)	0 (0.00%)	2 (3.64%)		2 (6.06%)	0 (0.00%)	2 (11.11%)		
Cirrhosis	Absent (%)	88 (79.28%)	42 (75.00%)	46 (83.64%)	0.350	26 (78.79%)	14 (93.33%)	12 (66.67%)	0.095	1.000
	Present (%)	23 (20.72%)	14 (25.00%)	9 (16.36%)		7 (21.21%)	1 (6.67%)	6 (33.33%)		
Bile duct invasion	Absent (%)	109 (98.20%)	55 (98.21%)	54 (98.18%)	1.000	32 (96.97%)	15 (100.00%)	17 (94.44%)	1.000	0.545
	Present (%)	2 (1.80%)	1 (1.79%)	1 (1.82%)		1 (3.03%)	0 (0.00%)	1 (5.56%)		
Cholangiectasis	Absent (%)	101 (90.99%)	50 (89.29%)	51 (92.73%)	0.742	29 (87.88%)	13 (86.67%)	16 (88.89%)	1.000	0.738
	Present (%)	10 (9.01%)	6 (10.71%)	4 (7.27%)		4 (12.12%)	2 (13.33%)	2 (11.11%)		
Lymph node metastasis	Absent (%)	98 (88.29%)	51 (91.07%)	47 (85.45%)	0.392	24 (72.73%)	12 (80.00%)	12 (66.67%)	0.458	0.050
	Present (%)	13 (11.71%)	5 (8.93%)	8 (14.55%)		9 (27.27%)	3 (20.00%)	6 (33.33%)		
Distant metastasis	Absent (%)	109 (98.20%)	56 (100.00%)	53 (96.36%)	0.243	32 (96.97%)	14 (93.33%)	18 (100.00%)	0.455	0.545
	Present (%)	2 (1.80%)	0 (0.00%)	2 (3.64%)		1 (3.03%)	1 (6.67%)	0 (0.00%)		
Tumor capsule	Absent (%)	1 (0.90%)	1 (1.79%)	0 (0.00%)	0.839	/	/	/	/	/
	Complete (%)	18 (16.22%)	9 (16.07%)	9 (16.36%)		/	/	/		
	Incomplete (%)	31 (27.93%)	17 (30.36%)	14 (25.45%)		/	/	/		
Tumor boundary	Explicit (%)	53 (47.75%)	25 (44.64%)	28 (50.91%)	0.529	12 (36.36%)	8 (53.33%)	4 (22.22%)	0.088	0.017
	Unclear (%)	18 (16.22%)	8 (14.29%)	10 (18.18%)		13 (39.39%)	3 (20.00%)	10 (55.56%)		
THPE	Absent (%)	89 (80.18%)	47 (83.93%)	42 (76.36%)	0.350	/	/	/	/	/
	Present (%)	22 (19.82%)	9 (16.07%)	13 (23.64%)		/	/	/		
Hemorrhage	Absent (%)	91 (81.98%)	44 (78.57%)	47 (85.45%)	0.460	31 (93.94%)	14 (93.33%)	17 (94.44%)	1.000	0.107
	Present (%)	20 (18.02%)	12 (21.43%)	8 (14.55%)		2 (6.06%)	1 (6.67%)	1 (5.56%)		
Tumor thrombus	Absent (%)	73 (65.77%)	38 (67.86%)	35 (63.64%)	0.692	26 (78.79%)	13 (86.67%)	13 (72.22%)	0.413	0.201
	Present (%)	38 (34.23%)	18 (32.14%)	20 (36.36%)		7 (21.21%)	2 (13.33%)	5 (27.78%)		

### Development, Performance, and Validation of the Radiomics Model

**Figure 2:**
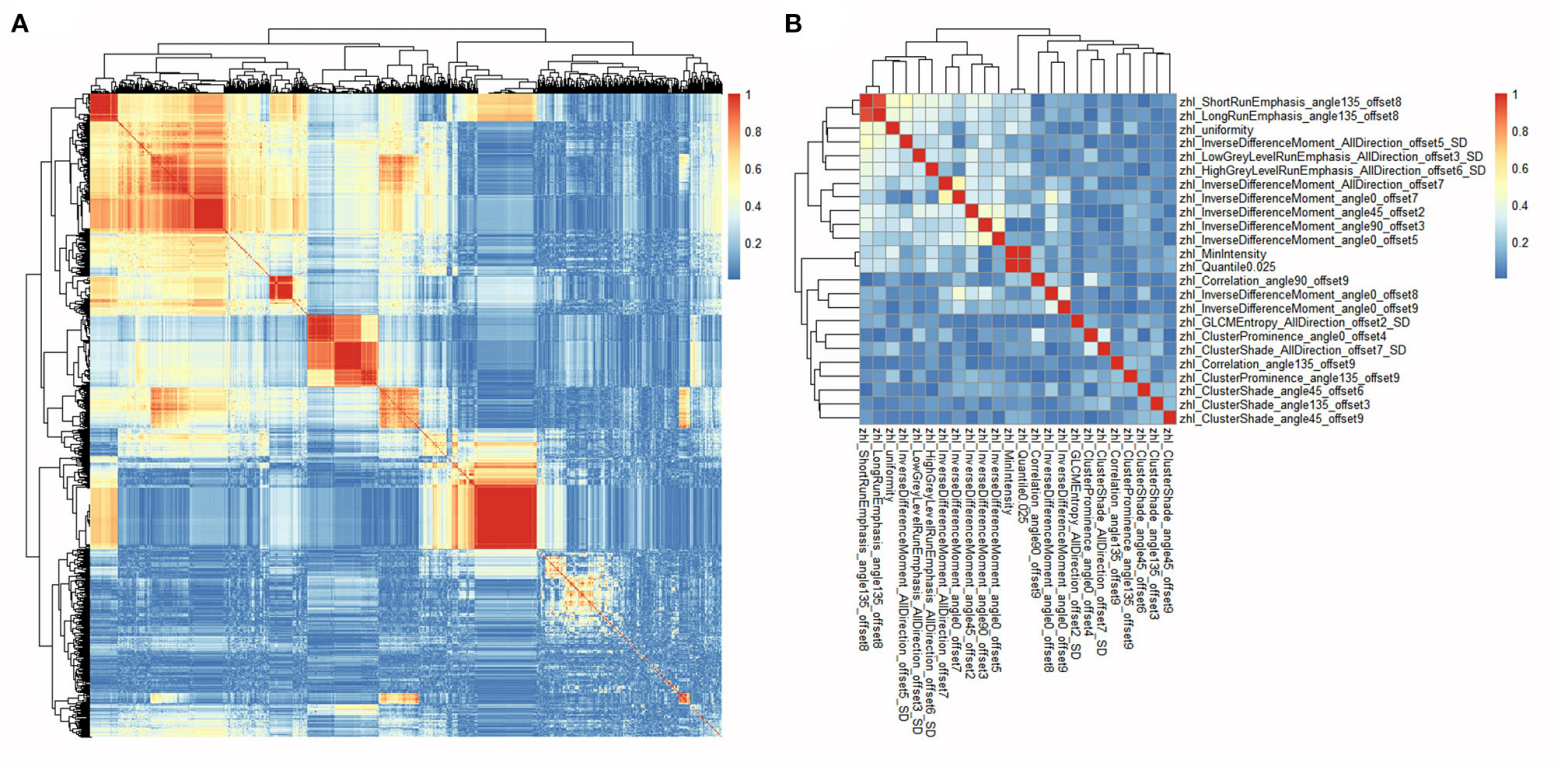
Heatmaps of correlations among radiomics features. **(A)** Heatmap depicting correlation coefficients matrix of 1,044 radiomics features in the training cohort. **(B)** Heatmap depicting correlation coefficients matrix of 24 selected radiomics features in the training cohort.

**Figure 3:**
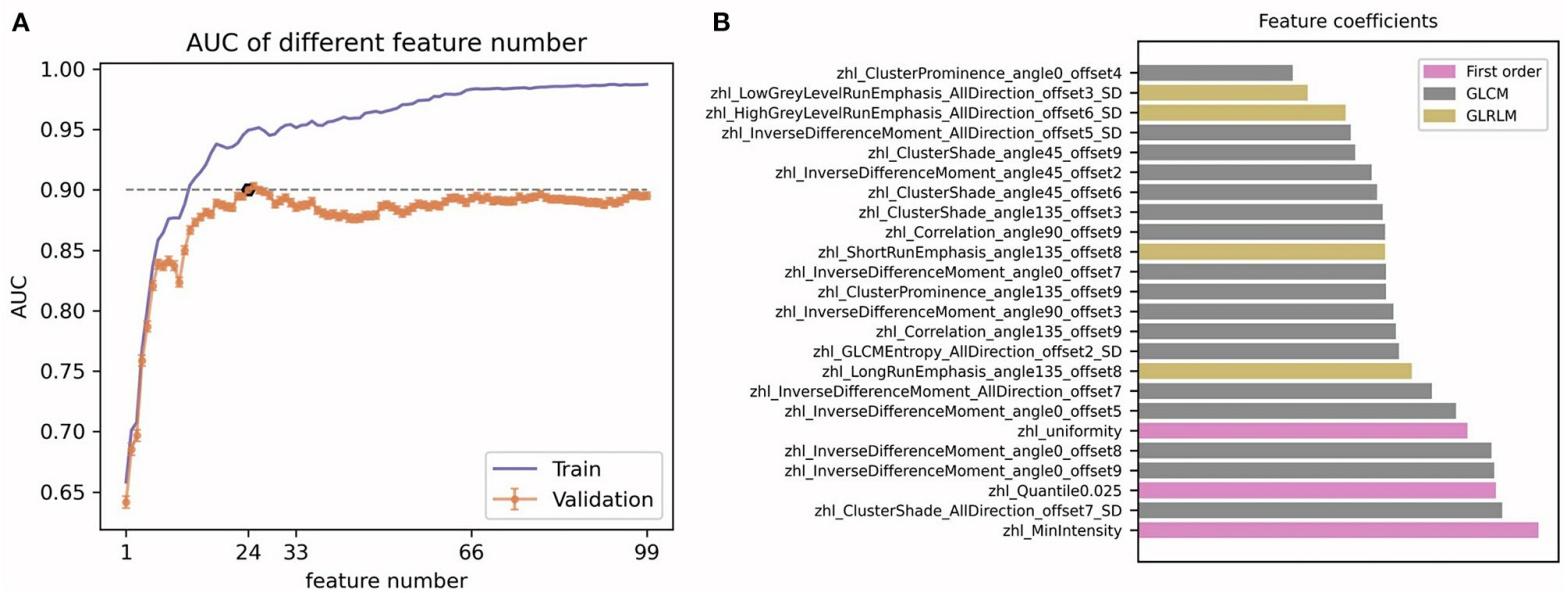
Radiomics features selection. **(A)** The determination of 24 radiomics features; **(B)** The ranks of the 24 selected features.

For each ROI of each patient, 1,044 radiomics features were extracted from the MRI image and then normalized. Pearson's correlation coefficients of a same feature of different ROIs from the same patient were calculated and all showed highly correlated with a value higher than 0.95. Thus, the average value of different ROIs for a same feature was calculated and used in subsequent analyses. Pearson's correlation coefficients of all 1,044 radiomics features in each patient were calculated (Figure 2) and highly correlated features were randomly eliminated, from which a total of 864 features were left. Subsequently, 24 radiomics features were selected based on the RFE method, including three first order histogram features, 4 GLRLM features, and 17 GLCM features (Figure 3). The list of these 24 features was shown in Supplementary Table 2. Pearson's correlation coefficients of 24 radiomics features were calculated and shown in Figure 2. The ranks of selected radiomics features were shown in Figure 3.

**Figure 4:**
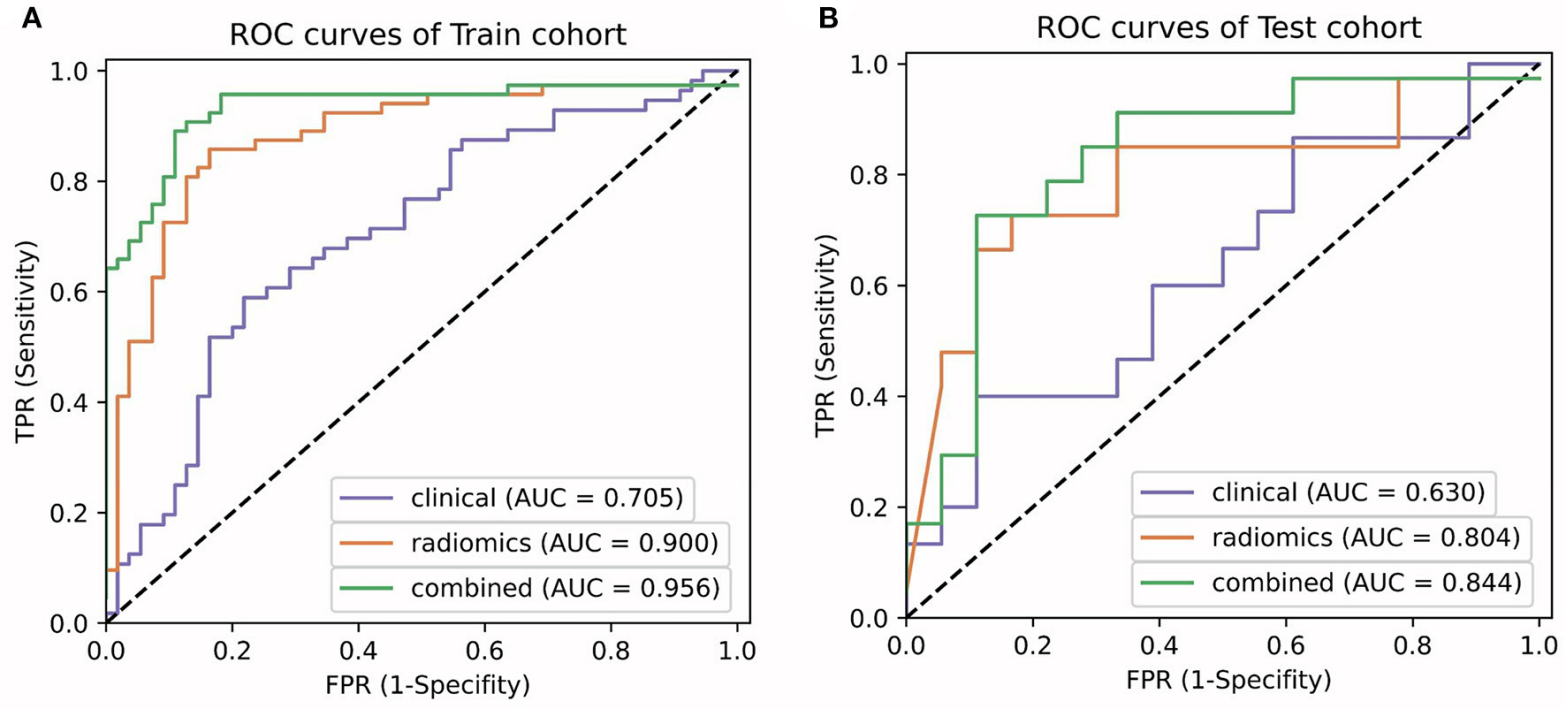
Comparison of ROC curves between the clinical model, radiomics model, and the LF model in the training **(A)** and test **(B)** cohorts.

A radiomics model that included these 24 features was then developed (Supplementary Materials), which showed satisfactory performance in the training cohort and the test cohort, with the AUCs of 0.900 (95% CI: 0.898–0.909) and 0.804 (95% CI: 0.792–0.845), respectively. The performance of the radiomics model in both cohorts was shown in Table 2 and Figures 4A,B.

**Table 2:** Performance of the radiomics model in the training cohort and the test cohort.

	**Training (*n* = 111)**	**Test (*n* = 33)**
AUC (95% CI)	0.900 (0.898–0.909)	0.804 (0.792–0.845)
Accuracy	0.856	0.727
Sensitivity	0.875	0.800
Specificity	0.836	0.667
PPV	0.845	0.667
NPV	0.868	0.800

### Development, Performance, and Validation of the Clinical Model and the Combined Model

Based on the univariate logistic regression analysis that included clinical features, height, viral hepatitis, splenomegaly, AST, PLT count, INR, ICG-R15, tumor size, and MELD score were identified and further included in the multivariate logistic regression analysis. The multivariate analysis found that PLT count and tumor size were independent risk factors for PHLF, with ORs of 0.990 (95% CI: 0.983–0.997) and 1.347 (95% CI: 1.139–1.594), respectively. The results of univariate and multivariate logistic regression analysis were shown in Table 3.

**Table 3:** Risk factors of PHLF in patients with HCC.

**Variables**	**Levels**	**Univariate logistic regression**			**Multivariate logistic regression**		
		**OR (95%CI)**	**Statistics**	***P*-value**	**OR (95%CI)**	**Statistics**	***P-*value**
Age	/	1.010 (0.977–1.045)	0.599	0.549			
Gender	Male	1	−1.164	0.244			
	Female	0.501 (0.157–1.604)					
Height	/	1.056 (0.993–1.124)	1.724	0.085	0.993 (0.916–1.076)	−0.167	0.867
Weight	/	1.015 (0.978–1.054)	0.808	0.419			
BMI	/	1.009 (0.894–1.138)	0.142	0.887			
DM	Absent	1	−0.033	0.974			
	Present	0.980 (0.296–3.248)					
Alcohol consumption	Absent	1	−0.479	0.632			
	Present	0.818 (0.359–1.862)					
NAFLD	Absent	1	−0.013	0.990			
	Present	0.982 (0.060–16.099)					
VH	Absent	1	1.911	0.056	1	−1.683	0.092
	HBV	2.625 (0.976–7.062)			0.017 (0.000–1.950)		
BCLC stage	0~A	1	/	/			
	B	1.369 (0.487–3.846)	0.596	0.551			
	C	1.244 (0.540–2.867)	0.607	0.607			
MELD score	/	1.252 (1.076–1.456)	2.911	0.004	2.062 (0.966–4.400)	1.871	0.061
ALBI score	/	3.179 (0.436–23.166)	1.141	0.254			
ICG-R15	/	1.009 (1.000–1.017)	2.071	0.038	1.007 (0.997–1.017)	1.411	0.158
TBIL	/	1.031 (0.985–1.080)	1.317	0.188	0.989 (0.924–1.059)	−0.306	0.760
Cr	/	1.007 (0.992–1.023)	0.906	0.365			
INR	/	570.563 (3.003–108,405.650)	2.371	0.018	1.758 (0.001–2,089.404)	0.156	0.876
AST	/	1.009 (0.999–1.020)	1.828	0.068	0.997 (0.977–1.018)	−0.291	0.771
ALT	/	1.006 (0.998–1.013)	1.476	0.140	1.006 (0.988–1.024)	0.657	0.511
GGT	/	1.001 (0.999–1.004)	1.050	0.294			
PLT	/	0.997 (0.993–1.001)	−1.458	0.145	0.990 (0.983–0.997)	−2.895	0.004
Tumor size	/	1.177 (1.057–1.311)	2.961	0.003	1.347 (1.139–1.594)	3.477	0.001
Number of tumors	1	1	/	/			
	2	1.200 (0.337–4.279)	0.281	0.779			
	>3	1.000 (0.409–2.444)	−1.73e-16	1.000			
Vascular invasion	Absent	1	0.332	0.740			
	Present	1.142 (0.521–2.503)					
Splenomegaly	Absent	1	1.479	0.139	1	−0.831	0.406
	Present	2.227 (0.771–6.436)			0.537 (0.124–2.329)		
Varicose veins	Absent	1	0.286	0.775			
	Present	1.200 (0.344–4.188)					
Adjacent tissue invasion	Absent	1	−0.001	1.000			
	Present	0.000 (0.000–inf)					
Cirrhosis	Absent	1	1.116	0.265			
	Present	1.704 (0.668–4.344)					
Bile duct invasion	Absent	1	−0.013	0.990			
	Present	0.982 (0.060–16.099)					
Cholangiectasis	Absent	1	0.630	0.529			
	Present	1.530 (0.407–5.750)					
Lymph node metastasis	Absent	1	−0.912	0.362			
	Present	0.576 (0.176–1.885)					
Distant metastasis	Absent	1	−0.001	1.000			
	Present	0.000 (0.000–inf)					
Tumor capsule	Complete	1	/	/			
	Incomplete	1.214 (0.379–3.888)	0.327	0.744			
Tumor boundary	Smooth	1	/	/			
	Rough	1.515 (0.663–3.464)	0.985	0.325			
THPE	Absent	1	−0.995	0.320			
	Present	0.619 (0.240–1.594)					
Hemorrhage	Absent	1	0.938	0.348			
	Present	1.602 (0.599–4.289)					
Tumor thrombus	Absent	1	−0.468	0.640			
	Present	0.829 (0.378–1.818)					

A clinical model that included PLT count and tumor size were constructed, while a combined model called the LF model that incorporated these two features and the radiomics score was constructed (Table 4). The performance of the clinical model and the LF model in both cohorts were shown in Table 5 and Figures 4A,B. The AUCs of the clinical model in the training cohort and the test cohort were 0.705 (95% CI: 0.700–0.716) and 0.630 (95% CI: 0.603–0.667), respectively. The AUCs of the LF model in the training cohort and the test cohort were 0.956 (95% CI: 0.955–0.962), and 0.844 (95% CI: 0.833–0.886), respectively. AUCs of both cohorts were significantly improved in the radiomics model, compared with those in the clinical model, which indicated the crucial role of radiomics features in predicting PHLF. Moreover, the LF model had the highest AUCs in both cohorts, which indicated it as a better prediction model for PHLF in HCC patients.

**Table 4:** Multivariate analysis of risk factors for the LF model.

**Variables**	**Levels**	**Multivariate logistic regression**		
		**OR (95%CI)**	**Statistics**	***P-*value**
Height	/	1.096 (0.882–1.360)	0.827	0.408
VH	Absent	1	−0.583	0.560
	HBV	0.151 (0.000–86.590)		
MELD score	/	1.020 (0.396–2.631)	0.041	0.967
ICG-R15	/	0.987 (0.965–1.010)	−1.095	0.273
TBIL	/	1.049 (0.951–1.156)	0.954	0.340
INR	/	3,513.303 (0.009–1,372,603,876.860)	1.243	0.214
AST	/	0.996 (0.952–1.043)	−0.155	0.877
ALT	/	1.002 (0.968–1.037)	0.114	0.910
PLT	/	0.973 (0.957–0.989)	−3.207	0.001
Tumor size	/	1.583 (1.119–2.241)	2.593	0.010
Splenomegaly	Absent	1	−1.554	0.120
	Present	0.083 (0.004–1.915)		
Radiomics score	/	1,697.575 (31.422–91,711.756)	3.860	0.000

**Table 5:** Performance of the clinical model and the LF model in the training cohort and the test cohort.

**Statistics**	**Clinical model**		**LF model**	
	**Training (*n* = 111)**	**Test (*n* = 33)**	**Training (*n* = 111)**	**Test (*n* = 33)**
AUC (95% CI)	0.705 (0.700–0.716)	0.630 (0.603–0.667)	0.956 (0.955–0.962)	0.844 (0.833–0.886)
Accuracy	0.685	0.667	0.901	0.788
Sensitivity	0.589	0.500	0.911	0.867
Specificity	0.782	0.889	0.891	0.722
PPV	0.733	0.526	0.895	0.722
NPV	0.652	0.643	0.907	0.867

## Discussion

In this study, we developed and validated the LF model that incorporated clinical and radiomics features for predicting PHLF in HCC patients, which had the best performance compared with the clinical model and the radiomics model. Although the clinical model included tumor size and PLT count could also predict PHLF in HCC patients, the performance was inferior to the radiomics model and the LF model. These results suggested that MRI images contained important information for liver function assessment and radiomics had huge potential in mining image information for PHLF prediction. Compared with other methods for liver function evaluation or prediction, our radiomics model has potential advantages in terms of convenience, effectiveness, and cost. Thus, the radiomics model could assist clinician in making treatment strategy.

Clinically, preoperative medical imaging is routinely performed for assessing tumor status and liver function in HCC patients. In fact, with the rapid development of medical imaging technology, it is increasingly important to obtain information on tumor areas and non-tumor areas from medical images. It has been shown that the FLR can be accurately measured before surgery by simulated resection assessment using 3D computed tomography (CT) imaging (24) and that the ratio of CT-derived liver volume (CTLV) to standard liver volume (SLV) can be used to predict the prognosis of acute liver failure (25). However, the physical volume of the liver does not always reflect liver function, which could be affected by blood supply, liver cirrhosis, and other factors (26). MRI is another valid and commonly used imaging method for assessing liver function preoperatively, especially dynamic hepatocyte-specific contrast-enhanced MRI (DHCE-MRI) with gadolinium-based contrast agents like Gd-EOB-DTPA. DHCE-MRI was found to be an ideal candidate for accurate determination of liver function before liver resection (27). Therefore, in the present study, we used Gd-EOB-DTPA-enhanced MRI as a valid preoperative imaging tool for PHLF risk assessment in HCC patients.

In recent years, due to the rapid development of related technologies, radiomics has become emergingly promising in medical research. Radiomics uses advanced computational methods to deeply explore the features of traditional images for cancer diagnosis, tumor staging, prognosis prediction, disease monitoring, and so on (28, 29). In this study, based on radiomics, we deeply mined potential risk factors of PHLF from preoperative MRI images of HCC patients and developed prediction models. The radiomics model had great performance in the training cohort and the test cohort, which included a total of 24 selected radiomics features (including three first order histogram features, 4 GLRLM features, and 17 GLCM features) that potentially reflected the features of the non-tumor area under the influence of tumor. The first order histogram features reflect overall differences between MRI images from a lower hierarchical level. Both the GLRLM features and the GLCM features are texture features, which had been previously reported to have potential values in HCC (14, 15). The GLRLM features are related to the grayscale distribution of the image, and the grayscale change of the image is indicative of the heterogeneity of the tissue. The GLCM features are a matrix describing the grayscale relationship between a pixel and its neighbors or pixels within a certain distance of a region. The GLCM features are further divided into five subclasses, including Cluster Prominence, Cluster Shade, Correlation, Joint Entropy, and Inverse Difference Moment (IDM). The Cluster Prominence reflects the abruptness of different tissues in MRI images and indicates abnormal features in liver and tumor tissues. The Cluster Shade is related to the symmetry of MRI images and suggests characteristic differences within normal liver tissue and between tumor and normal liver tissue. The IDM and Joint Entropy reflect the degree of regularity of the image texture. The lower the IDM, the higher the Joint Entropy, indicating the more irregular MRI image texture and the greater the tumor heterogeneity.

In our study, tumor size and PLT count were found to be independent clinical risk factors of PHLF. Although it is generally accepted that patients with large tumor size or multiple tumors can still be considered as candidates for surgical resection, they are prone to develop PHLF due to potentially insufficient FLR after extensive resection. Ma et al. studied 2,613 patients who underwent hepatectomy and found that the incidence of PHLF was significantly higher in the group with tumor diameter ≥ 50 mm than the group with tumor diameter <50 mm (8). Therefore, during the clinical management of HCC, tumor size should be accurately evaluated and considered to effectively prevent the occurrence of PHLF and the poor prognosis after resection. PLT count is one of the routine preoperative tests for surgical patients. PLT plays an important role in cooperating with hepatic sinusoidal endothelial cells and Kupffer cells to directly induce hepatocyte regeneration and improve liver function (30–33). Preoperative thrombocytopenia was reported to be an important independent predictor for the morbidity and mortality of postoperative complications (34). Thus, the need of additional perioperative care in patients with thrombocytopenia has also been proposed (35). Ohkohchi et al. identified the clinical impact of PLT transfusion by demonstrating that platelet transfusion improved liver function in patients with chronic liver disease (36, 37). In summary, insufficient PLT count is a significant risk factor for HCC patients, whose correction would bring about a significant reduction in the incidence of PHLF.

Our study had some limitations that need to be considered. Firstly, most included HCC patients in the current study were with hepatitis B, while only a few were with hepatitis C. In western countries, however, hepatitis C virus infection and alcoholic steatohepatitis are the main causes of HCC. Secondly, since only HCC patients who underwent hemihepatectomy were included, the remnant liver volume was not used as a risk factor. In fact, hepatectomy with less than half of the liver being resected would have a significantly lower risk of PHLF (9). Thirdly, relevant studies have also reported that intraoperative factors such as intraoperative bleeding, intraoperative blood transfusion, and hepatic portal block were related to PHLF (38, 39). As the aim of this study was to develop a preoperative prediction model for PHLF, intraoperative factors were not included. Fourthly, we used Gd-EOB-DTPA-enhanced MRI images in this study and the uptake of Gd-EOB-DTPA in HCC could be influenced by some factors, which need to be investigated in future studies. Finally, since this study was a retrospective study with a relatively small data set, the results need to be further validated in a large-scale prospective study.

In conclusion, our study, for the first time as we acknowledged, comprehensively evaluated radiomics features of MRI images in HCC patients and successfully established a radiomics-based clinical model for predicting PHLF, which could be potentially applied to assist treatment strategy development.

## Data Availability Statement

The original contributions presented in the study are included in the article/Supplementary Material, further inquiries can be directed to the corresponding author/s.

## Ethics Statement

The studies involving human participants were reviewed and approved by The First Affiliated Hospital of Sun Yat-sen University. Written informed consent for participation was not required for this study in accordance with the national legislation and the institutional requirements.

## Author Contributions

YC and ZL performed the study and drafted the manuscript. YM was involved in manuscript editing. BL and QZ performed data analysis. SP, SL, and MK designed the study. All authors read and approved the final manuscript.

## Conflict of Interest

The authors declare that the research was conducted in the absence of any commercial or financial relationships that could be construed as a potential conflict of interest.

## References

[1] Bray F Ferlay J Soerjomataram I Siegel RL Torre LA Jemal A. Global cancer statistics 2018: GLOBOCAN estimates of incidence and mortality worldwide for 36 cancers in 185 countries. CA Cancer J Clin. (2018) 68:394–424. 10.3322/caac.21492 30207593

[2] Mai RY Ye JZ Long ZR Shi XM Bai T Chen J. Preoperative aspartate aminotransferase-to-platelet-ratio index as a predictor of posthepatectomy liver failure for resectable hepatocellular carcinoma. Cancer Manag Res. (2019) 11:1401–14. 10.2147/CMAR.S186114 30863151 PMC6388945

[3] Prodeau M Drumez E Duhamel A Vibert E Farges O Lassailly G. An ordinal model to predict the risk of symptomatic liver failure in patients with cirrhosis undergoing hepatectomy. J Hepatol. (2019) 71:920–9. 10.1016/j.jhep.2019.06.003 31203152

[4] Tomimaru Y Eguchi H Gotoh K Kawamoto K Wada H Asaoka T. Platelet count is more useful for predicting posthepatectomy liver failure at surgery for hepatocellular carcinoma than indocyanine green clearance test. J Surg Oncol. (2016) 113:565–9. 10.1002/jso.24166 26751258

[5] Dasari BVM Hodson J Roberts KJ Sutcliffe RP Marudanayagam R Mirza DF. Developing and validating a pre-operative risk score to predict post-hepatectomy liver failure. HPB (Oxford). (2019) 21:539–46. 10.1016/j.hpb.2018.09.011 30361111

[6] Peng W Li JW Zhang XY Li C Wen TF Yan LN. A novel model for predicting posthepatectomy liver failure in patients with hepatocellular carcinoma. PLoS One. (2019) 14:e0219219. 10.1371/journal.pone.0219219 31269063 PMC6608969

[7] Longbotham D Young A Nana G Feltbower R Hidalgo E Toogood G. The impact of age on post-operative liver function following right hepatectomy: a retrospective, single centre experience. HPB (Oxford). (2020) 22:151–60. 10.1016/j.hpb.2019.06.014 31337601

[8] Ma KW Cheung TT She WH Chok KSH Chan ACY Dai WC. Risk prediction model for major complication after hepatectomy for malignant tumour - A validated scoring system from a university center. Surg Oncol. (2017) 26:446–52. 10.1016/j.suronc.2017.08.007 29113664

[9] Chin KM Allen JC Teo JY Kam JH Tan EK Koh Y. Predictors of post-hepatectomy liver failure in patients undergoing extensive liver resections for hepatocellular carcinoma. Ann Hepatobiliary Pancreat Surg. (2018) 22:185–96. 10.14701/ahbps.2018.22.3.185 30215040 PMC6125273

[10] Kauffmann R Fong Y. Post-hepatectomy liver failure. Hepatobiliary Surg Nutr. (2014) 3:238–46. 10.3978/j.issn.2304-3881.2014.09.01 25392835 PMC4207837

[11] Wang YY Zhao XH Ma L Ye JZ Wu FX Tang J. Comparison of the ability of Child-Pugh score, MELD score, and ICG-R15 to assess preoperative hepatic functional reserve in patients with hepatocellular carcinoma. J Surg Oncol. (2018) 118:440–5. 10.1002/jso.25184 30259515

[12] Zhang ZQ Xiong L Zhou JJ Miao XY Li QL Wen Y. Ability of the ALBI grade to predict posthepatectomy liver failure and long-term survival after liver resection for different BCLC stages of HCC. World J Surg Oncol. (2018) 16:208. 10.1186/s12957-018-1500-9 30326907 PMC6192221

[13] Wang L Xie L Zhang N Zhu W Zhou J Pan Q. Predictive value of intraoperative indocyanine green clearance measurement on postoperative liver function after anatomic major liver resection. J Gastrointest Surg. (2020) 24:1342–51. 10.1007/s11605-019-04262-5 31197694

[14] Chen S Zhu Y Liu Z Liang C. Texture analysis of baseline multiphasic hepatic computed tomography images for the prognosis of single hepatocellular carcinoma after hepatectomy: a retrospective pilot study. Eur J Radiol. (2017) 90:198–204. 10.1016/j.ejrad.2017.02.035 28583634

[15] Kiryu S Akai H Nojima M Hasegawa K Shinkawa H Kokudo N. Impact of hepatocellular carcinoma heterogeneity on computed tomography as a prognostic indicator. Sci Rep. (2017) 7:12689. 10.1038/s41598-017-12688-7 28978930 PMC5627280

[16] Romero-Gómez M Gómez-González E Madrazo A Vera-Valencia M Rodrigo L Pérez-Alvarez R. Optical analysis of computed tomography images of the liver predicts fibrosis stage and distribution in chronic hepatitis C. Hepatology. (2008) 47:810–6. 10.1002/hep.22112 18098299

[17] Barry B Buch K Soto JA Jara H Nakhmani A Anderson SW. Quantifying liver fibrosis through the application of texture analysis to diffusion weighted imaging. Magn Reson Imaging. (2014) 32:84–90. 10.1016/j.mri.2013.04.006 24239337

[18] Rahbari NN Garden OJ Padbury R Brooke-Smith M Crawford M Adam R. Posthepatectomy liver failure: a definition and grading by the International Study Group of Liver *Surgery* (ISGLS). Surgery. (2011) 149:713–24. 10.1016/j.surg.2010.10.001 21236455

[19] Pugh RN Murray-Lyon IM Dawson JL Pietroni MC Williams R. Transection of the oesophagus for bleeding oesophageal varices. Br J Surg. (1973) 60:646–9. 10.1002/bjs.1800600817 4541913

[20] Malinchoc M Kamath PS Gordon FD Peine CJ Rank J ter Borg PC. A model to predict poor survival in patients undergoing transjugular intrahepatic portosystemic shunts. Hepatology. (2000) 31:864–71. 10.1053/he.2000.5852 10733541

[21] Johnson PJ Berhane S Kagebayashi C Satomura S Teng M Reeves HL. Assessment of liver function in patients with hepatocellular carcinoma: a new evidence-based approach-the ALBI grade. J Clin Oncol. (2015) 33:550–8. 10.1200/JCO.2014.57.9151 25512453 PMC4322258

[22] Yushkevich PA Piven J Hazlett HC Smith RG Ho S Gee JC. User-guided 3D active contour segmentation of anatomical structures: significantly improved efficiency and reliability. Neuroimage. (2006) 31:1116–28. 10.1016/j.neuroimage.2006.01.015 16545965

[23] Lin X Yang F Zhou L Yin P Kong H Xing W. A support vector machine-recursive feature elimination feature selection method based on artificial contrast variables and mutual information. J Chromatogr B Analyt Technol Biomed Life Sci. (2012) 910:149–55. 10.1016/j.jchromb.2012.05.020 22682888

[24] Bégin A Martel G Lapointe R Belblidia A Lepanto L Soler L. Accuracy of preoperative automatic measurement of the liver volume by CT-scan combined to a 3D virtual surgical planning software (3DVSP). Surg Endosc. (2014) 28:3408–12. 10.1007/s00464-014-3611-x 24928235

[25] Tong C Xu X Liu C Zhang T Qu K. Assessment of liver volume variation to evaluate liver function. Front Med. (2012) 6:421–7. 10.1007/s11684-012-0223-5 23054504

[26] Hoekstra LT de Graaf W Nibourg GA Heger M Bennink RJ Stieger B. Physiological and biochemical basis of clinical liver function tests: a review. Ann Surg. (2013) 257:27–36. 10.1097/SLA.0b013e31825d5d47 22836216

[27] Rassam F Zhang T Cieslak KP Lavini C Stoker J Bennink RJ. Comparison between dynamic gadoxetate-enhanced MRI and (99m)Tc-mebrofenin hepatobiliary scintigraphy with SPECT for quantitative assessment of liver function. Eur Radiol. (2019) 29:5063–72. 10.1007/s00330-019-06029-7 30796575 PMC6682576

[28] Lambin P Leijenaar RTH Deist TM Peerlings J de Jong EEC van Timmeren J. Radiomics: the bridge between medical imaging and personalized medicine. Nat Rev Clin Oncol. (2017) 14:749–62. 10.1038/nrclinonc.2017.141 28975929

[29] Limkin EJ Sun R Dercle L Zacharaki EI Robert C Reuzé S. Promises and challenges for the implementation of computational medical imaging (radiomics) in oncology. Ann Oncol. (2017) 28:1191–206. 10.1093/annonc/mdx034 28168275

[30] Hirashita T Ohta M Iwashita Y Iwaki K Uchida H Yada K. Risk factors of liver failure after right-sided hepatectomy. Am J Surg. (2013) 206:374–9. 10.1016/j.amjsurg.2012.12.013 23835210

[31] Lesurtel M Clavien PA. Platelet-derived serotonin: translational implications for liver regeneration. Hepatology. (2014) 60:30–3. 10.1002/hep.27067 24700245

[32] Lisman T Porte RJ. Mechanisms of platelet-mediated liver regeneration. Blood. (2016) 128:625–9. 10.1182/blood-2016-04-692665 27297793

[33] Kurokawa T Ohkohchi N. Platelets in liver disease, cancer and regeneration. World J Gastroenterol. (2017) 23:3228–39. 10.3748/wjg.v23.i18.3228 28566882 PMC5434428

[34] Kaneko K Shirai Y Wakai T Yokoyama N Akazawa K Hatakeyama K. Low preoperative platelet counts predict a high mortality after partial hepatectomy in patients with hepatocellular carcinoma. World J Gastroenterol. (2005) 11:5888–92. 10.3748/wjg.v11.i37.5888 16270404 PMC4479695

[35] Bennett JJ Blumgart LH. Assessment of hepatic reserve prior to hepatic resection. J Hepatobiliary Pancreat Surg. (2005) 12:10–5. 10.1007/s00534-004-0950-3 15754093

[36] Maruyama T Murata S Takahashi K Tamura T Nozaki R Ikeda N. Platelet transfusion improves liver function in patients with chronic liver disease and cirrhosis. Tohoku J Exp Med. (2013) 229:213–20. 10.1620/tjem.229.213 23459612

[37] Takahashi K Murata S Ohkohchi N. Novel therapy for liver regeneration by increasing the number of platelets. Surg Today. (2013) 43:1081–7. 10.1007/s00595-012-0418-z 23180116

[38] Shiba H Ishida Y Wakiyama S Iida T Matsumoto M Sakamoto T. Negative impact of blood transfusion on recurrence and prognosis of hepatocellular carcinoma after hepatic resection. J Gastrointest Surg. (2009) 13:1636–42. 10.1007/s11605-009-0963-y 19582515

[39] Wakabayashi G Cherqui D Geller DA Buell JF Kaneko H Han HS. Recommendations for laparoscopic liver resection: a report from the second international consensus conference held in Morioka. Ann Surg. (2015) 261:619–29. 10.1097/SLA.0000000000001184 25742461

