# Supplementary material for: Prediction of Post-hepatectomy Liver Failure in Patients With Hepatocellular Carcinoma Based on Radiomics Using Gd-EOB-DTPA-Enhanced MRI: The Liver Failure Model
Source: Front Oncol. 2021 Mar 10;11:605296. doi: 10.3389/fonc.2021.605296 (PMC7987905; doi:10.3389/fonc.2021.605296)
Supplement: Supplementary file 1 [file Data_Sheet_1.docx]

Supplementary Material

# Supplementary Data

## Supplementary Materials and Methods

A same 3.0-T system (Siemens Healthineers) with eight-channel phased array coil was used to perform preoperative MRI examinations for all included HCC patients. The top to the lower edge of the liver was completely covered by the scanning scale. MRI scan sequences included half-Fourier single-shot turbo spin-echo (HASTE) sequence, fast low angle shot (FLASH) T1WI in/out of phase sequence imaging, FLASH T1WI fat suppression (FS) sequence axial imaging, and turbo spin-echo (TSE) T2WI navigation trigger axial imaging.

After as bolus injection of Gd-EOB-DTPA (Primovist®, 0.1 mL/kg body weight, 1 mL/s), images in arterial phase, portal phase, and hepatic venous phase were obtained by performing three-dimensional volume interpolated breathhold test (3D VIBE) T1WI (FS) sequence during suspended respiration at 30-35 s, 65-70 s, and 100-120 s, respectively. At 20 min after injection, additional hepatobiliary phase images were obtained. Specific parameters of each scan sequence are showed in Supplementary Table 1.

## Supplementary Results

**The Radiomics Model**

The probability is calculated with the following formula:

$$Probability=0.020*\mathrm{Intercept}-1.120*{SF}_{1}-0.921*{SF}_{2}-1.001*{SF}_{3}-0.730*{SF}_{4}+0.654*{SF}_{5}+0.714*{SF}_{6}+0.684*{SF}_{7}-0.433*{SF}_{8}+0.595*{SF}_{9}+0.889*{SF}_{10}-0.668*{SF}_{11}+0.822*{SF}_{12}+1.018*{SF}_{13}+0.693*{SF}_{14}+0.989*{SF}_{15}+0.996*{SF}_{16}+0.607*{SF}_{17}-0.691*{SF}_{18}+0.721*{SF}_{19}+0.694*{SF}_{20}-0.474*{SF}_{21}-0.581*{SF}_{22}+0.691*{SF}_{23}-0.766*{SF}_{24}$$

Where $SF_{n}=\frac{F_{n}-\overline{F_{n}}}{\sqrt{\sum_{\text{i}=1}^{m} F_{in}^{2}}}$, with m=24

And

F1=zhl_MinIntensity

F2=zhl_uniformity

F3=zhl_Quantile0.025

F4=zhl_GLCMEntropy_AllDirection_offset2_SD

F5=zhl_InverseDifferenceMoment_angle45_offset2

F6=zhl_InverseDifferenceMoment_angle90_offset3

F7=zhl_ClusterShade_angle135_offset3

F8=zhl_ClusterProminence_angle0_offset4

F9=zhl_InverseDifferenceMoment_AllDirection_offset5_SD

F10=zhl_InverseDifferenceMoment_angle0_offset5

F11=zhl_ClusterShade_angle45_offset6

F12=zhl_InverseDifferenceMoment_AllDirection_offset7

F13=zhl_ClusterShade_AllDirection_offset7_SD

F14=zhl_InverseDifferenceMoment_angle0_offset7

F15=zhl_InverseDifferenceMoment_angle0_offset8

F16=zhl_InverseDifferenceMoment_angle0_offset9

F17=zhl_ClusterShade_angle45_offset9

F18=zhl_Correlation_angle90_offset9

F19=zhl_Correlation_angle135_offset9

F20=zhl_ClusterProminence_angle135_offset9

F21=zhl_LowGreyLevelRunEmphasis_AllDirection_offset3_SD

F22=zhl_HighGreyLevelRunEmphasis_AllDirection_offset6_SD

F23=zhl_ShortRunEmphasis_angle135_offset8

F24=zhl_LongRunEmphasis_angle135_offset8

Cutoff: 0.4971

If Probability is ≥ Cutoff, the patient is predicted to develop PHLF.

# Supplementary Figures and Tables

## Supplementary Tables

**Supplementary Table 1. MRI scan sequence and parameters** (Chen et al., 2019)

| **Name** | **Orientation** | **Category** | **TR**  **(ms)** | **TE**  **(ms)** | **FOV**  **(mm)** | **Matrix** | **Reverse**  **angle** | | **Band**  **width** | **Seam thickness (mm)** | **Fat**  **saturation** | **Hold breath** |
| --- | --- | --- | --- | --- | --- | --- | --- | --- | --- | --- | --- | --- |
| T1WI IN/OUT PHASE | TRA | FLASH | 200 | 2.2/1.1 | 328×350 | 192×256 | | 65° | 930/977 | 6 | Yes/No | Yes |
| T1WI-FS | TRA | FLASH | 235 | 2.2 | 328×350 | 240×320 | | 70° | 822 | 6 | Yes | Yes |
| CONTRAST- Enhanced | | | | | | | | | | | | |
| T1WI | TRA | VIBE | 3.3 | 1.2 | 328×350 | 128×256 | | 13° | 501 | 2 | Yes | Yes |
| T2WI | TRA | TSE | 2000 | 75 | 328×350 | 240×320 | | 150° | 672 | 6 | Yes | No |
| T1WI (HBP) | TRA/ COR | VIBE | 3.3 | 1.2 | 328×350 | 154×256 | | 13° | 501 | 2 | Yes | Yes |

Abbreviations: TRA, transverse axial; COR, coronal; SAG, sagittal; FLASH, fast low angle shot; VIBE, volume interpolated breath-hold test; TSE, turbo spin echo; FS, fat suppression; HBP, hepatobiliary phase.

Chen, S., Feng, S., Wei, J., Liu, F., Li, B., Li, X., et al. (2019). Pretreatment prediction of immunoscore in hepatocellular cancer: a radiomics-based clinical model based on Gd-EOB-DTPA-enhanced MRI imaging. *Eur Radiol* 29(8)**,** 4177-4187. doi: 10.1007/s00330-018-5986-x.

**Supplementary Table 2. The selected 24 radiomics features**

| **No.** | **Features** |
| --- | --- |
| 1 | zhl_MinIntensity |
| 2 | zhl_uniformity |
| 3 | zhl_Quantile0.025 |
| 4 | zhl_GLCMEntropy_AllDirection_offset2_SD |
| 5 | zhl_InverseDifferenceMoment_angle45_offset2 |
| 6 | zhl_InverseDifferenceMoment_angle90_offset3 |
| 7 | zhl_ClusterShade_angle135_offset3 |
| 8 | zhl_ClusterProminence_angle0_offset4 |
| 9 | zhl_InverseDifferenceMoment_AllDirection_offset5_SD |
| 10 | zhl_InverseDifferenceMoment_angle0_offset5 |
| 11 | zhl_ClusterShade_angle45_offset6 |
| 12 | zhl_InverseDifferenceMoment_AllDirection_offset7 |
| 13 | zhl_ClusterShade_AllDirection_offset7_SD |
| 14 | zhl_InverseDifferenceMoment_angle0_offset7 |
| 15 | zhl_InverseDifferenceMoment_angle0_offset8 |
| 16 | zhl_InverseDifferenceMoment_angle0_offset9 |
| 17 | zhl_ClusterShade_angle45_offset9 |
| 18 | zhl_Correlation_angle90_offset9 |
| 19 | zhl_Correlation_angle135_offset9 |
| 20 | zhl_ClusterProminence_angle135_offset9 |
| 21 | zhl_LowGreyLevelRunEmphasis_AllDirection_offset3_SD |
| 22 | zhl_HighGreyLevelRunEmphasis_AllDirection_offset6_SD |
| 23 | zhl_ShortRunEmphasis_angle135_offset8 |
| 24 | zhl_LongRunEmphasis_angle135_offset8 |
